# Diabetes Mellitus and Its Implications in Aortic Stenosis Patients

**DOI:** 10.3390/ijms22126212

**Published:** 2021-06-09

**Authors:** Laura Mourino-Alvarez, Nerea Corbacho-Alonso, Tamara Sastre-Oliva, Cecilia Corros-Vicente, Jorge Solis, Teresa Tejerina, Luis R. Padial, Maria G. Barderas

**Affiliations:** 1 Department of Vascular Physiopathology, Hospital Nacional de Paraplejicos (HNP), SESCAM, 45071 Toledo, Spain; lmourino@sescam.jccm.es (L.M.-A.); ncorbacho@sescam.jccm.es (N.C.-A.); tsastre@sescam.jccm.es (T.S.-O.); ceciliacorros@yahoo.com (C.C.-V.); 2 Department of Cardiology, Hospital Universitario 12 de Octubre and Instituto de Investigación Sanitaria Hospital 12 de Octubre (imas12), 28041 Madrid, Spain; 3 Atria Clinic, 28009 Madrid, Spain; 4 Centro de Investigación Biomédica en Red de Enfermedades Cardiovasculares (CIBERCV), Instituto de Salud Carlos III, 28029 Madrid, Spain; 5 Department of Pharmacology, School of Medicine, Universidad Complutense, 28040 Madrid, Spain; teje@med.ucm.es; 6 Department of Cardiology, Hospital Virgen de la Salud, SESCAM, 45004 Toledo, Spain; lrpadial@gmail.com

**Keywords:** diabetes mellitus, aortic stenosis, calcification, risk prediction

## Abstract

Aortic stenosis (AS) and diabetes mellitus (DM) are both progressive diseases that if left untreated, result in significant morbidity and mortality. Several studies revealed that the prevalence of DM is substantially higher in patients with AS and, thus, the progression from mild to severe AS is greater in those patients with DM. DM and common comorbidities associated with both diseases, DM and AS, increase patient management complexity and make aortic valve replacement the only effective treatment. For that reason, a better understanding of the pathogenesis underlying both these diseases and the relationships between them is necessary to design more appropriate preventive and therapeutic approaches. In this review, we provided an overview of the main aspects of the relationship between AS and DM, including common comorbidities and risk factors. We also discuss the established treatments/therapies in patients with AS and DM.

## 1. Introduction

Aortic stenosis (AS), of which Degenerative is the most common form (DAS), is the valvular heart disease that most often requires surgical intervention in developed countries [1]. The development of DAS is progressive and it shares risk factors with atherosclerosis in its early stages. Clinical and histological data indicate that far from being a passive degenerative process, DAS is an active disease that involves the deposition of lipoproteins, chronic inflammation and, in later stages, the osteoblastic transformation of interstitial cells (ICs) in association with the formation of calcium deposits in valve tissue, leading to calcific AS [2]. There are many similarities between the early inflammatory stages of this disease and atherosclerosis [3]. Likewise, the progression of the disease is primarily regulated by valve ICs (VICs), the most abundant cell type in the aortic valve (AV). In fact, varying degrees of VIC activation have been found in calcified AS valves, including phenotypes that resemble those of the myofibroblasts and osteoblasts responsible for calcium deposition in the valve. As a result of this deposition, the stiffness of the AV leaflets increases and there is a narrowing of the AV that affects its haemodynamics.

Hypertension, hypercholesterolemia and diabetes mellitus (DM) are among the diseases/factors seen to influence the progression of both atherosclerosis and DAS. Both type 1 and type 2DM (T1DM and T2DM) accelerate the development of atherosclerosis, which is not only due to the hyperglycaemia produced, but also, to the associated insulin resistance, dyslipidaemia, etc. In addition, T2DM induces a marked inflammatory response and increased lipid accumulation. These mechanisms also affect the development of DAS and they are associated with hypertrophic left ventricular remodelling, increased left ventricle mass, left ventricle end-systolic dimension and reduced systolic function. All these processes indicate that DM has an adverse effect on cardiac function and, indeed, AS patients with DM have been seen to have significantly worse left ventricle diastolic function [4]. Therefore, DM directly impairs both myocardial diastolic and systolic functions, and it indirectly affects cardiac function by inducing comorbidities such as coronary artery disease. It is through these mechanisms that DM increases the risk of Heart Failure (HF) in patients with AS.

DAS is typically asymptomatic for long periods, up to several years, and from the time of diagnosis disease progression is unavoidable, evolving rapidly with a poor prognosis. Thus, finding new therapeutic options to treat this disease is one of its greatest challenges today. As DM influences the survival of DAS patients, careful risk stratification is required to adopt the best strategy to manage each patient. If we also take into account that there is no single parameter that allows us to adequately predict disease evolution, all the information available must be considered when taking decisions in clinical practice. Despite all the efforts to date, the management of DAS is complex as the only effective treatment is aortic valve replacement through either a surgical (SAVR) or transcatheter (TAVI) intervention [5]. Recent information indicates the convenience of performing surgery in patients with severe asymptomatic DAS [6]. Moreover, T2DM is a risk factor in patients with DAS undergoing surgery, affecting both the survival and quality of life (QoL) of these patients [7]. Thus, regular medical examinations, including electrocardiogram and imaging techniques (echocardiogram, cardiac-MRI or Cardiac-CT), are necessary to monitor the evolution of DAS in patients with DM, and to define the most appropriate time for treatment and strategy.

In terms of pharmacological treatments, studies have been carried out, both with statins, and with other drugs related to calcium and phosphate metabolism, yet there are still no therapies capable of slowing down or altering calcification [8]. Valve calcification has been linked with immunological phenomena that would favour the mineralization of VICs, the main components of the AV, such as an increase in interleukin 6 (IL-6) and TNF-alpha expression. Hence, therapies directly targeting the immune response in DAS could be an appropriate strategy to manage this chronic condition. As for specific therapies targeting diabetic patients with DAS, oral anti-diabetic drugs or insulin that might target the AV or myocardium can be used. In theory, this therapy should halt DAS progression, reduce its hemodynamic repercussions on left ventricular (LV) function and remodelling, and consequently improve clinical outcomes [9].

Both DM and AS commonly occur in the elderly population, and while there is evidence that the prevalence of DM is substantially greater in patients with AS, the full effects of DM on the long-term prognosis of patients with AS remains unclear. Some cross-sectional observational studies revealed that DM is associated with an increased risk of AV calcification [10,11], yet opinions as to whether DM accelerates the progression of AS [12,13] or not vary [1]. As a result, here we review the evidence in the literature of a relationship between diabetes and DAS, including studies on the prevalence of DM in patients with AS and the rate of progression from mild to severe AS in these patients. In addition, we consider different therapies administered to DM patients and their effect on DAS progression, as well as different options for valve replacement in patients with DAS and concomitant DM. Finally, we briefly comment on other important comorbidities that affect these patients.

## 2. Diabetes Mellitus as an Important Risk Factor of Aortic Stenosis

Nowadays, it seems clear that DAS and DM are associated, yet the prevalence of diabetes in the DAS population varies greatly in different studies. DAS was reported in 41% of diabetics [14], although this number is around 30% in other studies [15,16]. Indeed, the SALTIRE study reported AS in only 5% of DM patients [17] and, likewise, there is little consensus regarding the relative prevalence of diabetes in the general population and in DAS patients. In 2003, 20% of severe AS patients were diabetics as opposed to 18% in the age-matched controls [18]. When comparing individuals with or without changes in AV structure, there were differences in the prevalence of diabetes (3.8 and 1.3%, respectively), although individuals with AS were significantly older and, thus, there was also a higher prevalence of arterial hypertension, obesity, diabetes, hypercholesterolemia and cardiovascular disease in this group [19]. Alternatively, studies on larger cohorts indicate that the prevalence of diabetes is notably higher in patients with AS. In the Multi-Ethnic Study of Atherosclerosis (MESA), with 5723 participants, DM was shown to increase the odds of developing AS (OR 2.06; 95% CI, 1.39–3.06) [20]. Moreover, the Cardiovascular Health in Ambulatory Care Research Team (CANHEART) conducted a population-based observational study on a cohort of 9.8 million adults and, after a 13-year median follow-up period, they concluded that DM was associated with greater risk of AS (HR—Hazard ratio, 1.49; 95% CI, 1.44–1.54) [21]. There are also studies into the effect of diabetes on the QoL, progression of calcification and survival of patients with AS. Most of them indicate that DM influences the event-free survival (EFS) of patients with AS, both those who were treated conservatively and those subjected to percutaneous or surgical intervention, although this remains somewhat controversial. For example, in the MESA study, DM was not associated with AS progression among participants with calcification at baseline [20], and in the Helsinki Aging Study DM was not an independent predictor of AV calcification [11]. By contrast, DM was found to be an independent predictor of poor outcome after intervention [22] and an independent determinant of cardiovascular mortality in patients with severe AS [23]. In addition, diabetes was recently identified as an independent predictor of AS-related events [24]. In this interesting study, a CURRENT-AS risk score was devised that integrated different independent predictors of AS related events 1 year after diagnosis in asymptomatic patients with severe AS, which included DM, haemodialysis, any concomitant valve disease, Left Ventricle Ejection Fraction (LVEF) < 60%, haemoglobin ≤ 11 g/dL and chronic lung disease.

The existing controversies may reflect the different characteristics of the cohorts examined in each study, with different basal clinical characteristics and nationalities. Nationalities are important as different cultures and lifestyles affect the prevalence of DM. In fact, the leading three risk factors for diabetes are high body mass index (BMI), dietary risks and ambient particulate matter pollution [25]. Data from these studies should also consider that the global prevalence of DM increased from 211.2 million in 1990 to 476.0 million in 2017, a 129.7% increase [25].

## 3. Role of Diabetes Mellitus in the Calcification of Aortic Stenosis

**Figure 1:**
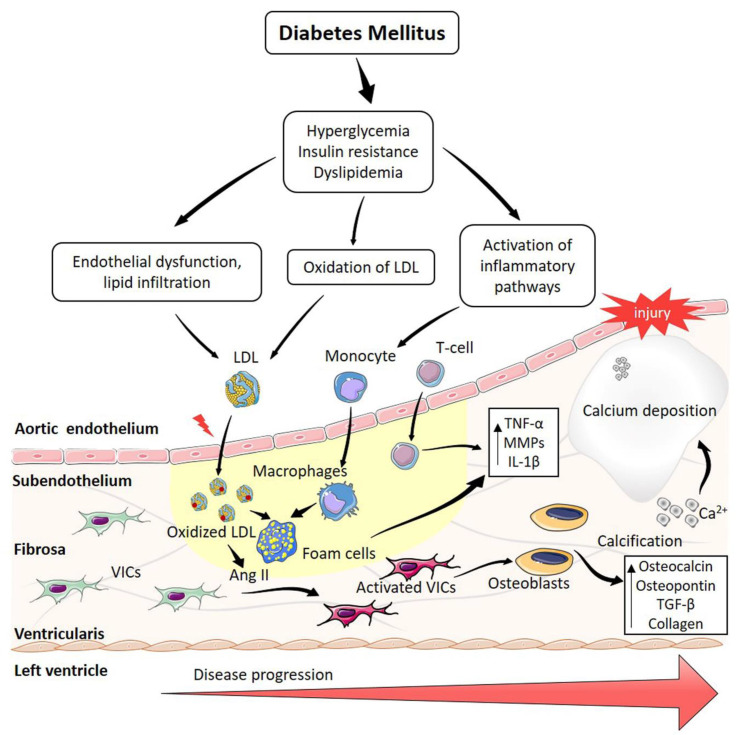
Scheme of the pathophysiological connection of diabetes mellitus and aortic stenosis. Hyperglycemia, insulin resistance and dyslipidemia trigger several pathogenic processes such as endothelial dysfunction, oxidation of low-density lipoproteins (LDL) and inflammation. This activates valve interstitial cells (VICs) and produces the remodelling of the extracellular matrix. Osteoblast-like cells produce calcium crystals that finally result in a macrocalcification. AngII, angiotensin II; TNF, Tumor necrosis factor; MMPs, matrix metalloproteinases; IL, interleukin; TGF, transforming growth factor.

DAS is now considered an active process with AV calcification representing a terminal event. Various pathogenic processes are now recognised as key drivers of AV stenosis and mineralization [9], such as lipid infiltration/retention, inflammation and osteogenesis (Figure 1). Insulin resistance and T2DM can play an important role in the development of AV mineralization and in the progression of AS [10,26]. Assessing AV calcification by computed tomography in 6780 MESA participants with metabolic syndrome revealed a linear and graded relationship between AV calcification and the number of metabolic syndrome components, both in men and women (*p* < 0.001 for both) [9]. Furthermore, insulin resistance was associated with left ventricular hypertrophy and the progression of AS in patients with hypercholesterolemia [10]. The putative mechanisms in which DM favours vascular calcification include activation of AKT by O-Linked *N*-acetylglucosamine [27] and altered levels of small leucine-rich proteoglycans [28,29], although these mechanisms require further investigation in DAS.

In a murine model genetically modified to produce DAS, the dysmetabolic state of T2DM, as well as a diabetogenic diet [30,31], contributed to early mineralization of the AV and calcified AV pathogenesis, findings that reflect the clinical association observed between type T2DM and calcified AS. DM augments the expression of proinflammatory CRP and tissue factor in patients with severe AS [32], which is consistent with the central role of inflammation in the pathogenesis of both glucose disorders—DM and AS [33,34,35,36,37,38]. To evaluate the inflammation and calcification in patients with and without DM, surgically explanted AVs with severe AS were analysed. AVs from DM patients were seen to be more mineralized, with stronger expression of osteogenic markers such as Runx2 and ALP, while inflammation was similar in both populations [39]. By contrast, the AV area was greater and the mean pressure gradient lower in DM patients, which were classified with a clinically less advanced disease. These results suggest that DM patients could be in a more advanced disease stage of severe AS, with a higher grade of mineralization than non-DM patients, and requiring valve replacement.

## 4. Comorbidities Common to Type2 Diabetes Mellitus and Degenerative Aortic Stenosis

**Figure 2:**
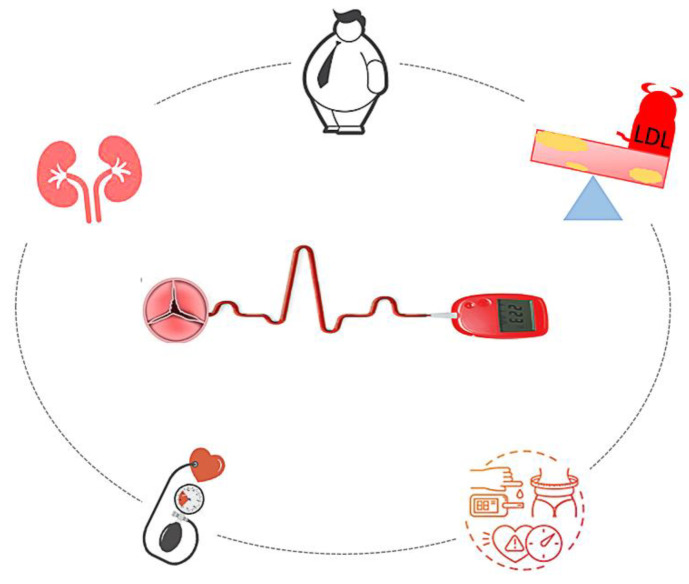
Common comorbidities in Type 2 diabetes mellitus and degenerative aortic stenosis. A high percentage of patients with severe DAS or T2DM had at least one or more comorbidities, some of which are common in patients with DAS and T2DM, such as hypertension, dyslipidemia, atherosclerosis, obesity, CHD and metabolic syndrome.

The coexistence of two or more pathological conditions, defined as multimorbidity, is a key priority for global health. The prevalence of multimorbidity has been estimated to be 55–98%, and it is associated with poorer outcomes in terms of disease management and treatment decisions [40,41]. Accordingly, there is a need to improve the follow-up of such patients, which represents an important public health challenge. Identifying common patterns of multimorbidity will help improve the QoL of these patients, as well as reduce the use and costs of health services [42]. Such comorbidities, including chronic kidney disease (CKD) and coronary artery disease, are common in DM patients, and they affect risk–benefit analyses because they have an independent influence on the patient’s life expectancy, regardless of the valve disease. Therefore, the effect of DM on AS patients can be compounded by common comorbidities. Importantly, a high percentage of patients with severe DAS or T2DM had at least one or more comorbidities [43,44], some of which are common in patients with DAS and T2DM, such as hypertension, dyslipidemia or obesity [43,45] (Figure 2). This situates patients with both DAS and T2DM at a higher risk of suffering an adverse event.

### 4.1. Hypertension

Hypertension involves pathophysiological mechanisms such as the upregulation of the renin–angiotensin–aldosterone system (RAAS), endothelium dysfunction/oxidative stress, activation of the sympathetic nervous system (SNS), as well as activation of the immune system [46]. Hypertension is common in patients with DM and it has been associated with more rapid progression of DAS [47]. In T1DM, hypertension is related to diabetic nephropathy, whereas in patients with T2DM it is associated with insulin resistance and atherosclerosis. The presence of hypertension in diabetic patients, as well as in DAS, triggers adverse cardiovascular events, which involve macrovascular and microvascular complications such as myocardial infarction, coronary artery disease, stroke or nephropathy [48,49]. Moreover, the SEAS (Simvastatin Ezetimibe in Aortic Stenosis) study associated hypertension with a 56% higher rate of ischemic cardiovascular events and higher mortality relative to normotensive patients with DAS [50]. Although there are no studies about specific antihypertensive treatments in patients with DAS, correct control and optimal treatment of hypertension in these patients is crucial to reduce cardiovascular risk and prevent cardiovascular complications [51].

### 4.2. Chronic Kidney Disease

Chronic kidney disease (CKD) is a very prevalent condition worldwide (9.1% of the global population) [52] and there is a high prevalence of DAS in patients with CKD, in which calcification progresses faster than in patients with normal renal function [53]. Moreover, the presence of CKD in DAS patients increases “all cause” and cardiovascular mortality, and these patients have a higher perioperative mortality after AV replacement [47]. However, AV replacement is also associated with a strong reduction in mortality at 5 years regardless of the CKD stage [54]. Alternatively, approximately 20–40% of diabetic patients develop kidney disease, one of the most common complications of diabetes [55]. Moreover, diabetic nephropathy contributes to a higher risk of cardiovascular disease and mortality [56], with a need for glycaemic control and good management of cardiovascular risk factors to reduce such complications and mortality in diabetic patients.

### 4.3. Dyslipidemia and Atherosclerosis

Dyslipidemia is well established as a cardiovascular risk factor, and it has been estimated that more than 50% of the adult population worldwide has dyslipidemia [57]. Dyslipidemia causes lipid deposition in the arteries that promotes atherosclerosis, sharing pathogenic mechanisms with the initial stages of DAS [9,58]. In addition, several studies have demonstrated a relationship between lipoprotein levels and the risk of developing DAS and calcium deposition [59,60,61]. By contrast, dyslipidemia and diabetes commonly occur together, accelerating atheroma formation in DM patients due to hyperglycaemia and insulin resistance [62]. Moreover, insulin resistance not only plays a role in the progression of atherosclerosis, but also in the progression of hypertension and dyslipidemia [63], thus increasing the risk of AS. Although experimental models suggest that lipid-lowering therapy with statins might prevent the progression of AS, three retrospective clinical studies failed to identify such benefits [51].

### 4.4. Obesity

Obesity is a multifactorial disease, and it is considered to be another factor that could increase the risk of AS. Although there are studies that produced conflicting results [64,65,66], a relationship between the risk of developing DAS and BMI or waist circumference has been proposed [67]. Moreover, a recent study concluded that in terms of human genetic obesity, there was an association with the risk of DAS and replacement surgery [68]. A high BMI is also a risk factor for DM and more than 90% of patients with DM are overweight or obese [69]. Obesity is worse when associated with insulin resistance and it is involved in complications of DM [70]. As described previously, DM on its own contributes to faster progression of DAS and, thus, the combination of obesity and DM complicates the risk of AS.

### 4.5. Metabolic Syndrome

Metabolic syndrome involves the accumulation of several risk factors for cardiovascular diseases and DM [71]. Metabolic syndrome, obesity and DM are interrelated, and they lead to cardiovascular complications. The principal mechanisms underlying metabolic syndrome are abdominal adiposity and insulin resistance [72]. These alterations occur simultaneously, increasing the risk of adverse events and leading to the association of metabolic syndrome with the progression of DAS irrespective of traditional risk factors [73]. Furthermore, experimental models have demonstrated that metabolic syndrome is associated with mechanical and structural changes of the AV and early calcification [74]. Alternatively, there are reports that patients with metabolic syndrome manifest a greater risk of developing DM [75]. Thus, improvements in metabolic syndrome status are needed to reduce the risk of DM and, with it, the risk of DAS.

Finally, additional comorbidities in DAS and DM increase the risk of complications, affecting patient management. Thus, a multifactorial approach is necessary to diminish the frequency of adverse events and to establish adequate treatment strategies.

## 5. The Importance of Risk Prediction in AS Patients

Risk prediction in AS patients remains an important challenge to cardiologists, especially for asymptomatic patients. Current guidelines generally recommend a conservative strategy based on careful observation, waiting until indications for AV replacement emerge [51,76]. Nevertheless, asymptomatic patients with severe AS usually end up developing symptoms and, furthermore, their rate of EFS is only 30% to 50% after 2 years. Importantly, the status of the ventricles and pulmonary circulation of these patients may be irreversible affected, and they are at higher risk of AV-related death and sudden death [77,78,79]. Moreover, valve deterioration is accelerated in individuals with DM, such that the timing of intervention should be carefully defined in these patients [80].

Hence, it is important to improve risk scores that define potentially high-risk groups that could benefit from other strategies or treatments. To date, risk stratification is mainly based on clinical characteristics such as age and cardiovascular risk factors, imaging studies such as transthoracic echocardiogram and cardiac magnetic resonance imaging (cMRI), and stress testing [51]. Severe comorbidities should be also taken into account by clinicians, although they are not included in risk scores per se. Serum biomarkers are also, in general, excluded from these scores, and only the elevation of serum B-type natriuretic peptide (BNP) is mentioned in the current guidelines as a predictor of risk and taken into account when recommending surgery to asymptomatic patients [51,81,82]. Although high levels of BNP and troponin I have been associated with the presence of severe autonomic failure in patients with AS, future studies must evaluate their true prognostic value [83].

Currently, risk prediction scores for AS do not include metabolomic and proteomic data, although these approaches provide biochemical profiles focused on the characterization of the products of gene transcription. In fact, metabolites and protein biomarkers are powerful tools in managing different diseases, including errors of metabolism in newborns [84], cancer [85], kidney disease [86], or other cardiovascular diseases such as heart failure [87] and acute coronary syndromes (ACS) [88]. For example, the diagnosis of T2DM is based on glucose and haemoglobin A1c levels. There has been little research into AS biomarkers, although some proteomic studies have been carried out using animal models and different human samples, such as AV tissue or plasma [89]. Several metabolomics studies have been carried out in recent years and some of these even combined metabolomics and proteomics, highlighting the involvement of inflammation, lipid metabolism, coagulation and haemostasis, extracellular matrix remodelling, and oxidative stress [90,91]. Several studies analysed plasma and urine to define molecular profiles of the disease [92,93], or to obtain information about the effects of valve replacement [94], yet without obtaining prognostic information. A comprehensive metabolomics study was recently published, in which 106 stenotic valves were analysed, demonstrating that the levels of lysophosphatidic acid were correlated with faster hemodynamic progression, permitting patients at risk of a faster progression rate to be selected [95]. Unfortunately, despite the promise of these studies, none of the defined indicators have been used in larger clinical cohorts with different kinds of patients (asymptomatic, symptomatic, in association with risk factors, etc.), and these should be studied further to evaluate the utility of these proteins. Importantly, T2DM was analysed as a further cardiovascular risk factor in these studies into AS, although populations with and without this pathology have yet to be compared. It will be interesting to perform such studies as T2DM is a polygenic metabolic disease in which behavioural and environmental factors may play a significant role, as well as affecting AV tissue and other affected organs. As circulating proteins can be influenced by these factors, their study will provide a more complete biological readout of these disease markers [96]. It is important to note that the complications of T2DM and its associated healthcare costs can be dramatically reduced by effective prevention and treatment. As such, it is encouraging to think that defining prognostic markers and accurate risk prediction scores may slow down the course of AS in DM patients, thus improving their management.

## 6. The Management of Patients with DAS and DM

### 6.1. Treatment/Therapies: Cardiac Effects during Glycaemic Control

In T2DM patients, the association between poor glycaemic control and cardiovascular events is a well-established fact. Long-term glucose control reduces 25% of microvascular events in T2DM but it does not affect macrovascular events (myocardial infarction (MI) and stroke). Several drugs have been developed to achieve effective glycaemic control in T2DM [97]. In fact, current guidelines recommend a combination of glucose-lowering drugs to achieve HbA1c targets, which usually represents a challenge for physicians, especially in patients with concomitant heart disease. Indeed, most of the commonly used anti-diabetic drugs are contraindicated in HF patients and, thus, there is an urgent need for an oral agent capable of improving glycaemic control while providing cardiovascular benefits. Currently, clinical trials involving glucagon-like peptide-1 receptor antagonists (GLP-1RA: liraglutide, luraglutide and semaglutide) and sodium-glucose cotransporter-2 (SGLT2) inhibitors (SGLT2-is: empagliflozin, canagliflozin, dapagliflozin and ertugliflozin) are the only ones of interest for cardiovascular purposes. SGLT2 proteins are responsible for roughly 90% of filtered glucose reabsorption [98,99,100,101]. SGLT2-is function by reducing renal tubular glucose reabsorption, thereby reducing blood glucose without stimulating insulin release as their action is independent of β-cell function. Thus, SGLT2-is may be a useful option in obese and hypertensive patients since they increase lipolysis and fatty acid oxidation, producing weight loss and providing anti-hypertensive benefits due to their natriuretic effect [97,102].

### 6.2. SGLT2 Inhibitors and Cardiovascular Outcomes

SGLT2-is delay the progression of microvascular changes, thus affecting T2DM patients and improving arterial stiffness. The recent EMPAREG OUTCOME trial proposed a major therapeutic breakthrough in the treatment of T2DM patients [103]. Patients with a high cardiovascular risk (47% with a history of MI and 25% with a history of stroke) were randomized to a treatment with empagliflozin or a placebo, in addition to standard medical care. The trial was prematurely terminated due to the magnitude of the cardiac benefits. Specifically, empagliflozin significantly reduced the primary composite end-point of cardiovascular mortality, non-fatal MI and non-fatal stroke by 14% (HR 0.86; (0.74–0.99), *p* = 0.04 for superiority). The secondary end-points were also significantly reduced in the patients that received empagliflozin, with a 38% relative risk reduction (RRR) for cardiovascular death, a 32% RRR for death from any cause and a 35% RRR for HF-related hospitalization. Furthermore, empagliflozin is the first glucose-lowering agent that significantly reduces HF hospitalization and slows renal disease progression in DM patients. In a large, randomized trial [104] that included over 7000 patients, empagliflozin produced a 39% RRR for incident/worsening nephropathy and a 44% RRR for doubling serum creatinine levels relative to the placebo.

Despite the remarkable cardiac benefits of SGLT2-is, their underlying mechanism of action remains unclear. It seems that the metabolic effects are likely to be responsible for the reduction in cardiovascular mortality and HF hospitalization, improved glycaemic control, or decreased body weight and blood pressure. Moreover, tight glycaemic control previously failed to reduce either mortality or HF [4]. It is more likely that these benefits are due to hemodynamic effects, specifically reduced blood pressure and decreased extracellular volume [105]. In a recent study, empagliflozin was shown to ameliorate diastolic function in a non-diabetic HF porcine model, mitigating histological and molecular remodelling, and reducing both left ventricle and cardiomyocyte stiffness [106]. These cardiac benefits of empagliflozin seem to be mediated by a shift in myocardial metabolism away from glucose towards cardiac utilization of ketone bodies (KB), free fatty acids (FFAs), and branched chain amino acids (BCAAs), thus improving myocardial energetics and cardiac function. These findings highlight the therapeutic potential of empagliflozin for HF, even in non-diabetic patients, and they warrant further investigation [106,107].

Canagliflozin, another SGLT2-is, significantly reduced the primary composite end-point of cardiovascular mortality, non-fatal MI, and non-fatal stroke by 14% in the CANVAS program [103]. Empagliflozin and canagliflozin had similar effects in preventing HF hospitalization (a RRR of 35% and 33%, respectively), although, unlike empagliflozin [103], canagliflozin did not reduce the secondary end-point of cardiovascular mortality (HR 0.87 (0.74–1.01), *p* = 0.06) [10]. Like the EMPA-REG OUTCOME, canagliflozin did not affect the rate of MI or stroke. The recent DECLARE-TIMI58 trial demonstrated that dapagliflozin reduced the co-primary end-point of cardiovascular death and HF hospitalization by 17%, which reflected a lower rate of hospitalization for HF (HR 0.73; (0.61–0.88)) [108]. Hence, the cardiac benefits are probably a class effect of the SGLT2-is pharmacological family. Importantly, the CANVAS trial showed a significant increase in below-the-knee amputations (6.3 vs. 3.4 participants per 1000 patient-years) [109]. However, the recent OBSERVE-4D meta-analysis [12] concluded that canagliflozin does not increase amputations and that it has a similar safety profile to other SGLT2-is [110].

**Figure 3:**
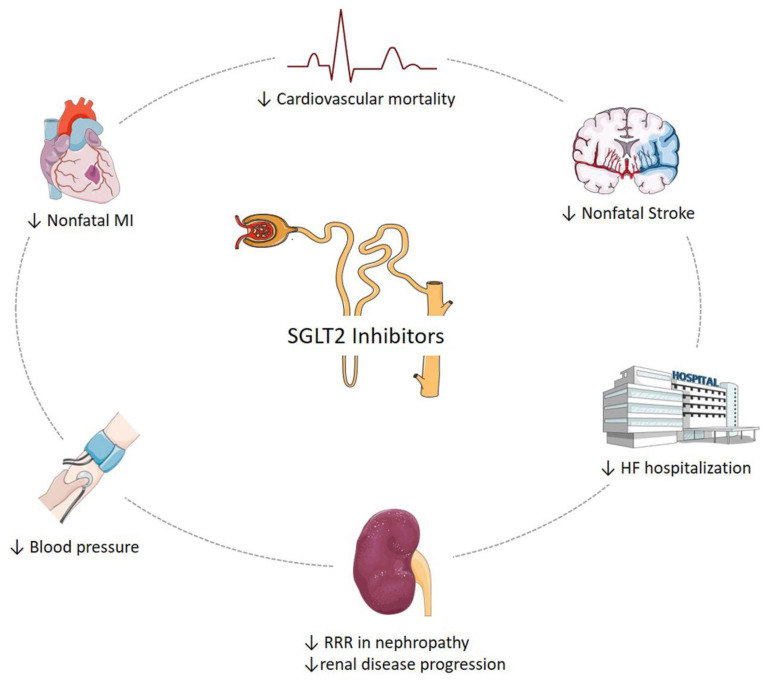
SGLT2 Inhibitors and Cardiovascular Outcome. SGLT2-is have significantly improved cardiac outcomes in T2DM patients and they seem to show a class effect, with empagliflozin being the first glucose-lowering agent that significantly reduces HF, mortality, hospitalization and renal disease progression in diabetic patients.

In summary, SGLT2-is are the latest class of oral anti-hyperglycaemic agents available to treat patients with T2DM. Their novel mechanism of action makes these medications an intriguing option for patients throughout the natural history of T2DM and as a possible adjunct therapy for T1DM with close supervision. SGLT2-is have demonstrated non-inferiority and additional metabolic benefits, thus preserving and enhancing Beta-cell function. The clinical trials involving SGLT2-is have significantly improved cardiac outcomes in T2DM patients and they seem to show a class effect, with empagliflozin being the first glucose-lowering agent that significantly reduces HF, mortality, hospitalization and renal disease progression in diabetic patients (Figure 3).

The professional organizations that guide the management of diabetes are the American Diabetes Association (ADA), the European Association for the Study of Diabetes (EASD), the American Association of Clinical Endocrinology/American College of Endocrinology (AACE/ACE), and the Endocrine Society and American College of Physicians (ACP). All these organizations agree on individualizing therapy through a patient-centred approach to guide the choice of medication. Based on recent findings from cardiovascular outcome trials, the consensus report guides healthcare professionals as to how to manage hyperglycaemia in patients with atherosclerotic cardiovascular disease, CKD and HF. The consensus also considers patients without atherosclerotic cardiovascular disease (ASCVD) or CKD, and the need to minimize hypoglycaemia and weight gain, thereby promoting weight loss. The ADA and AACE/ACE published updated recommendations for the management of patients with T2DM in their latest guidelines (2018). Moreover, the European Society of Cardiology, in collaboration with the EASD, released updated guidelines for diabetes, prediabetes and heart disease in 2019, recommending SGLT2-is and GLP-1 agonists as first-line therapies for people with diabetes who have heart disease or are at risk of heart disease [111]. Although there are many side effects, including the recently identified episodes of ketoacidosis related to SGLT2-is use, this class of agents may be a good option to treat diabetic patients.

### 6.3. Treatment and Prognosis of AS in Patients with DM: The Impact of DM after Trans-Catheter AV Implantation

Treatment of diabetic patients with severe AS using anti-diabetic medication, in the form of both oral tablets and insulin, may target the valve or the myocardium. In theory, specific medical therapy should halt AS progression, reduce its haemodynamic repercussions on LV function and remodelling, and improve clinical outcomes. However, none of the current medications used in patients with AS to treat cardiac disease or comorbidities appear to affect the survival of patients with AS, nor have they been proven to slow down the course of the disease [9]. The only treatment shown to improve survival in severe AS patients is AV replacement, which, in diabetic patients, causes elevated post-operative morbidity and mortality relative to non-diabetic patients [112]. These results were consistent whether the trans-femoral or trans-apical approach was used. In addition, DM impairs LV mass regression after AV replacement, which indicates that people with diabetes have less benefits and are at greater risk of AV replacement than non-diabetic patients [113]. However, a large retrospective study recently showed that T2DM patients have lower intrahospital mortality after AV replacement than non-diabetic patients [114].

Continuous improvements in surgical approaches and techniques, including minimally invasive AV replacement and increased use of new tissue (stented or sutureless) valves, might to some extent explain the conflicting results regarding AV replacement in diabetic patients. In a careful review of the literature surrounding the outcome of SAVR or TAVI interventions in diabetic patients, the analysis in the PARTNER trial showed that diabetic patients have a lower all-cause mortality after TAVI than after SAVR [115]. In the TAVIK prospective, single-centre registry, DM was not identified as an independent factor associated with reduced survival after TAVI. Nevertheless, insulin-treated patients were seen to have a significantly lower survival rate than patients with orally treated DM and those without DM at 1 year (75.7% vs. 84.5% vs. 84.7%: pairwise *p* < 0.01) and 3 years (56.9% vs. 65.9% vs. 67.9%: adj. *p* < 0.05) after TAVI. However, insulin-treated diabetes was not identified as an independent risk factor for higher mortality in the 1st (HR 1.29; 95% CI 0.97–1.72, *p* = 0.084) and 3rd years (HR 1.21; 95% CI 0.98–1.49; *p* = 0.079) after multivariable adjustment [116]. Similarly, DM was not associated with a higher 30-day or 12-month all-cause mortality after TAVI, nor was it associated with decreased post-procedural QoL [117]. Moreover, diabetic patients did not have a worse short-term outcome compared to non-diabetic patients with the use of TAVI or SAVR [118,119]. By contrast, it was shown that TAVI was associated with a higher 1-year mortality in a multivariable model (HR 1.30; 95% CI 1.13–1.49; *p* < 0.001). This mortality was more strongly associated with insulin treated diabetics (HR 1.57; 95% CI 1.28–1.91; *p* < 0.001) than non-insulin-treated diabetics (HR 1.17; 95% CI 1.00–1.38; *p* = 0.052) [120].

Despite the many studies referred to above, the optimal treatment for severe AS in both non-diabetic and diabetic patients remains unclear. As such, several trials are currently investigating the early/elective interventional approaches to treat asymptomatic severe AS patients.

## 7. Conclusions

At present, the data have revealed an association between AS and DM, and that DM has a detrimental effect on the QoL and survival of AS patients. Despite the continuing search for novel therapeutic approaches to date, the only effective treatment is aortic valve replacement. More studies are needed to identify approaches that will help delay the progression of these diseases, thus improving the prognosis and evolution of patients with AS and DM.
